# Toxicological Impact and *in Vivo* Tracing of Rhodamine Functionalised ZIF-8 Nanoparticles

**DOI:** 10.3389/ftox.2022.917749

**Published:** 2022-07-01

**Authors:** Prateek Goyal, Pushpanjali Soppina, Superb K. Misra, Eugenia Valsami-Jones, Virupakshi Soppina, Swaroop Chakraborty

**Affiliations:** ^1^ Materials Engineering, Indian Institute of Technology Gandhinagar, Gandhinagar, India; ^2^ Biotechnology and Bioinformatics, Sambalpur University, Burla, India; ^3^ Biological Engineering, Indian Institute of Technology Gandhinagar, Gandhinagar, India; ^4^ School of Geography, Earth and Environmental Sciences, University of Birmingham, Birmingham, United Kingdom

**Keywords:** metal organic frameworks, fluorescent label, nanoparticles tracing, nanotoxicity assessment, MOFs toxicity, ZIF-8 MOFs

## Abstract

Metal Organic Frameworks (MOFs) are extensively used for a wide range of applications due to their exceptionally high surface area. MOF particles are conventionally in micron size, but the nanosized MOFs show good transportation/mobility due to their small size, and when combined with the high surface area of MOFs, it makes MOF nanoparticles an ideal candidate to study for environmental remediation. Therefore, it is important to study the ecotoxicological impact of these MOFs. In this study, we developed rhodamine labelled nanoparticles of zinc imidazolate metal organic framework (ZIF-8 MOFs) as a means of *in vivo* tracing the MOF translocation in *C. elegans*. Rhodamine B isothiocyanate functionalized ZIF-8 MOFs nanoparticles (RBITC@ZIF-8 MOF nanoparticles; size 44 ± 7 nm) were fed to the worms naturally within a concentration range of 0.16–16.4 μg mg^−1^. Fluorescence was detected in the pharyngeal and gut lumen regions of the worms after 4 h of treatment, for exposure concentrations >0.163 μg mg^−1^. A higher intensity of fluorescence was observed at the end of 24 h for all exposure concentrations. Worms treated with RBITC@ZIF-8 MOF concentrations of ≥1.63 μg mg^−1^ for 24 h showed a bright stable fluorescence signal at the tail region. The uptake of RBITC@ZIF-8 MOF for an exposure concentration of 0.163, 1.63, and 8.2 μg mg^−1^ was found to be 52.1, 11.4 and 28.6%, respectively. Through this study, we showed that RBITC@ZIF-8 MOFs can be exposed to *C. elegans* and imaged at low concentrations of ∼0.16 μg mg^−1^.

## 1 Introduction

Metal Organic Frameworks (MOFs) are an emerging class of porous and crystalline materials that are extensively used in environmental (catalysis, energy, adsorption, gas capture) and biological applications (drug delivery). These classes of new-age materials form structures through a process of self-assembly of complex subunits centred around transition metals and interconnected with several polyfunctional organic ligand molecules (Furukawa et al., 2013; Kajal et al., 2022). The dominant characteristics of MOFs include low density, high surface area and porosity, wide flexibility and tunability of shape, structure and pore sizes (Goyal et al., 2022). The ligands present in MOFs are organic molecules and can be made of pyridyl group, tricarboxylic acids, and imidazole groups. There are various families of MOFs based on the kind of metal nodes and organic linkers used in the manufacturing process, with wide-ranging physicochemical properties. Zeolitic Imidazolate Frameworks (ZIFs) are among the family of MOFs, which are topologically isomorphic with zeolites. These MOFs have extraordinary thermal and chemical stability, high microporosity and a large surface area. Among the various classes of ZIFs, ZIF-8, which has Zn^2+^ present as a metal cation linked with the organic ligand 2-methylimidazole, has been used extensively. The exceptionally large surface area 962 m2/g and good thermochemical stability of ZIF-8 MOFs (Abdelhamid, 2021; Kajal et al., 2022; Cravillon et al., 2009) have made them an ideal candidate for environmental remediation applications. The tailored design of ZIF-8 MOF responses to various stimuli, e.g., pH, singlet oxygen and redox potential gradient also make them a promising material for drug delivery and biomedical applications (Feng et al., 2021; Hoseinpour and Shariatinia, 2021). However, exposure of these ZIF-8 MOFs to the environment poses a risk of leaching Zn ions into the environment, which could act as a potential environmental hazard. To be more specific, the ZIF-8 constituting zinc has been demonstrated to have a higher level of toxicity compared to other metals such as zirconium and iron, with the majority of the toxicity caused due to the ability of Zn^2+^ to alter cellular metabolism leading to cell damage (Tamames-Tabar et al., 2014). Additionally, the small particulate size of ZIF-8 allows more extensive binding of cellular protein due to higher surface area and consequently enhanced alteration in the cellular function and cell toxicity (Wallace et al., 2007). The toxicological profile of ZIF-8 MOFs largely remains unclear. Although ZIF-8 MOFs are reported for their potential toxicity, there is a lack of significant exploration of the way they behave when exposed to any bio-ecotoxicological model organisms. It is therefore important to understand the environmental risk of ZIF-8 MOFs before they become widely used in environmental remediation applications.

*Caenorhabditis elegans (C. elegans)* serves as a model organism to study the toxicological potential of materials, because of its well-known and relatively simple cellular and molecular biology (Gonzalez-Moragas et al., 2015; Wu et al., 2019). Zhou et al. (2016) demonstrated that the accumulation of silica nanoparticles occurred in the intestine and pharyngeal regions of the worms. In a recent article, we reported dual labelled nanoprobes for quantification and imaging studies in *C. elegans*. These nanoprobes were effective even at very low concentrations (0.033 μg mg^−1^) and were used for *in vivo* toxicity assessment (Butreddy et al., 2022).

The objective of this study is to analyse the *in vivo* effects of ZIF-8 MOF nanoparticles labelled with RBITC in *C. elegans*, which was achieved by measuring the uptake, accumulation and biodistribution of RBITC-ZIF-8 MOF in *C. elegans*. The worms were naturally fed with a mixture of *E. coli* OP50 bacteria and RBITC-ZIF-8 MOF nanoparticles in a ratio of 1:1 after a starvation period of 1 h. The low natural background level of zinc in *C. elegans*, allowed us to detect the accumulated concentration of RBITC-ZIF-8 MOF nanoparticles in the worms by measuring the non-enriched zinc level post-exposure to RBITC-ZIF-8 MOF nanoparticles. The fluorescent labelling technique helped to determine the bioaccumulation of RBITC-ZIF-8 MOF nanoparticles and also study the consequence of the exposure on the egg-laying capacity or survival rate compared to untreated worms. The fluorescent labelling approach of ZIF-8 MOF nanoparticles along with the *C. elegans* model presents a promising strategy for screening the ecotoxicity potential of metal organic framework (MOF) nanoparticles.

## 2 Experimental Methods

### 2.1 Materials

All chemicals used in this study were of analytical grade. 2-Methylimidazole and Rhodamine B isothiocyanate (RBITC) dye was procured from Sigma-Aldrich. Zinc nitrate hexahydrate (Zn (NO_3_)_2_.6H_2_O) was procured from HiMedia Laboratories and methanol was procured from Merck.

### 2.2 Synthesis of ZIF-8 MOF and Fluorescent Labelled ZIF-8 MOF Nanoparticles

The synthesis of ZIF-8 MOF nanoparticles was performed following the methodology of Cravillon et al. (2009), with a slight modification. Zn (NO_3_)_2_.6H_2_O (0.05 M, 100 ml) and 2-Methylimidazole (0.4 M, 100 ml) solutions were prepared separately in methanol. The Zn (NO_3_)_2_.6H_2_O solution was added rapidly to the 2-Methylimidazole solution under stirring conditions. The mixture turned turbid (milky white) after continuous stirring for 1 h. The obtained suspension was then washed multiple times using methanol to remove unreacted salts. The precipitate was dried in a vacuum oven for 4 h at 60°C. The recovered powder was activated at 120°C under vacuum to remove trapped solvent molecules. 2 mg of RBITC dye was added to 20 mL of ethanol and stirred for 20 min. ZIF-8 MOF solution was prepared by sonicating 125 mg of ZIF-8 MOF powder in 30 mL of ethanol (Time: 10 min and Amplitude: 30%). ZIF-8 MOF suspension was added dropwise to the above prepared RBITC dye solution under stirring. After 6 h of continuous stirring, the resulting RBITC@ZIF-8 MOF suspension was repeatedly washed and sonicated until the untagged dye was removed completely.

### 2.3 Characterisation of MOF Nanoparticles

ZIF-8 MOF nanoparticles and RBITC@ZIF-8 MOF nanoparticles were subjected to a range of physicochemical characterisations. X-ray diffraction analysis (Bruker D8 Discover, Cu-K_α_ source, 40 kV, 30 mA, 2θ range: 20°–80°) of ZIF-8 MOF nanoparticles was performed to identify the phase purity and crystallinity of the material. Transmission Electron Microscopy (TEM, FEI, Themis 60–300) and Scanning Electron Microscopy (SEM, JEOL JSM7900F) analysis were performed on the MOF nanoparticles to understand the morphology and size distribution. The size distribution of ZIF-8 MOF nanoparticles was calculated using ImageJ software. Fourier Transformed Infrared Spectroscopy (FTIR, Perkin Elmer spectrum 2) analysis on the ZIF-8 MOF nanoparticles was performed (4,000 cm^−1^ to 400 cm^−1^) to investigate the molecular fingerprint of the samples. The attachment of RBITC dye on ZIF-8 MOF nanoparticles was confirmed by investigating the characteristic peaks in the FTIR spectrum. The confirmation of untagged RBITC dye removal was investigated by UV-Visible spectral scanning in the range of 200–900 nm (Perkin Elmer, Lambda 365). The stability of RBITC tagging on the ZIF-8 MOF nanoparticles was investigated by time-dependent fluorescence spectroscopy (Horiba Jobin Fluorolog-3) for up to 72 h. The excitation and emission wavelength of RBITC dye were taken as 507 nm and 568 nm respectively. The dye attachment on the ZIF-8 MOF nanoparticles was also confirmed by using a Nikon Eclipse Ti2-E microscope. 10 µL of RBITC@ZIF-8 MOF nanoparticles was smeared on the glass slide and were imaged under epifluorescence illumination.

Zeta Potential and hydrodynamic size measurement of both variants of MOF nanoparticles were performed using Dynamic Light Scattering (DLS, Malvern Nano ZS, United Kingdom). The dissolution profile of ZIF-8 MOF and RBITC@ZIF-8 MOF nanoparticles were performed at a concentration of 50 μg mL^−1^ in S-buffer for 24 h. The particles were suspended in S-buffer and at each time point, aliquot was collected and subjected to ultrafiltration at 6,000 rpm for 5 min (MWCO: 3 kDa, Amicon, Merck) (Chakraborty and Misra, 2019). The filtrate at each time point was collected and digested using 2% HNO_3_. The concentration of dissolved Zn was measured using Inductively Coupled Plasma-Optical Emission Spectroscopy (ICP-OES, Avio 200, Perkin Elmer, United States).

### 2.4 Tracing RBITC@ZIF-8 MOF Nanoparticles *in vivo*

#### 2.4.1 Feeding MOF Nanoparticles

For brood size and longevity assays, ten synchronized L4 stage worms were starved for 1h on a Nematode Growth Media (NGM) plate without OP50 bacteria before nanoparticle treatment. These starved worms were exposed to a range of concentrations of RBITC@ZIF-8 MOF nanoparticles (0.163, 1.63, 4.1, 8.2, 16.4 μg mg^−1^) in S-buffer containing *E. coli* OP50 bacteria at 1:1 ratio. The MOF nanoparticles treatment for each concentration was performed at 20°C for 4 h and 24 h. There are no predicted environmental concentration data available for Zn-MOFs. Therefore, we started with the PEC values available for ZnO nanoparticles. An estimation through modelling shows that the environmental concentration of ZnO nanoparticles ranges from 0.24 to 0.661 μg kg^−1^ in soil and sediments, which is currently showing an increasing trend going up to 1.82 μg kg^−1^ (Wu et al., 2019). We hence chose the exposure concentration range between 1 mg L^−1^ to 50 mg L^−1^. Since the exposure data is normalised to mass of *C. elegans,* we have reported the exposure concentration to be between 0.163 and 1.63 μg per milligram of C.*elegans*.

#### 2.4.2 Imaging

L4 stage worms were fed with RBITC@ZIF-8 MOF nanoparticles at different concentrations (0.163–16.4 μg mg^−1^). For control, worms were treated with S-buffer and RBITC dye (0.163 μg mg^−1^) alone. Post 4 and 24 h of exposure, worms were transferred onto a glass coverslip with an agar pad and paralyzed using 1 mM Levamisole in S-buffer. Imaging was acquired under epifluorescence illumination with 10X, 0.45 NA objective using a fully motorized Nikon Eclipse Ti2-E microscope equipped with an EM-CCD camera (Andor iXonUltra 897) controlled by NIS-Elements Advanced Research image acquisition software.

#### 2.4.3 Brood Size and Life Span Analysis Post-Exposure

Synchronized L4 stage worms were treated for 4 h with RBITC@ZIF-8 MOF nanoparticles (0.163–16.4 μg mg^−1^), Zn^2+^ (3.27 μg mg^−1^), imidazole (16.4 μg mg^−1^), and RBITC (0.163 μg mg^−1^). Control worms were treated only in S-buffer. Treated worms were transferred to a fresh NGM plate seeded with OP50 bacteria and maintained at 20°C. For brood size, every day treated worms were transferred to a new plate and then counted for progenies. In parallel, we also counted alive and active treated worms to score their life span after exposure to the RBITC@ZIF-8 MOF nanoparticles.

#### 2.4.4 Estimation of Zn Uptake

∼15,000 synchronized L4 stage worms for each treatment condition were collected and washed with S-buffer by centrifugation at 1,300 *g* for 2 min. Pelleted worms are treated with RBITC@ZIF-8 MOF nanoparticles (0.163–8.2 μg mg^−1^) diluted in S-buffer in a 1:1 ratio to get the desired concentration. Two sets of control conditions were set up, one with only S-buffer and the other with Zn^2+^ alone (3.27 μg mg^−1^). After 24 h of exposure, worms were washed with S-buffer and spun down at 1,300 g for 2 min. Pelleted worms were frozen at −80°C followed by lyophilization for 48 h (McColl et al., 2012; Ganio et al., 2016). The lyophilized worms were weighed and then digested with the required volume of ashing mixture containing 30% H_2_O_2_ and 69% HNO_3_ in a 3:1 ratio at 100°C until the worms were digested giving a clear solution. The natural background concentration and uptake of RBITC@ZIF-8 MOF nanoparticles in *C. elegans* were measured using Inductively Coupled Plasma Mass Spectroscopy (ICP-MS, Nexion 2000; Perkin Elmer, USA). The estimation of Zn in control and RBITC@ZIF-8 MOF nanoparticles exposed samples were performed in Helium KED mode (gas flow = 5 mL min^−1^, sweeps/reading = 40, Dwell time = 40 ms). LOD and LOQ for the desired method for Zn analysis were calculated to be 0.11 and 0.34 μg L^−1^ respectively. for The equation used for calculating the net uptake/accumulation of the RBITC@ZIF-8 MOF nanoparticles was:

$$Uptake=\frac{C_{t}}{C_{e}}$$ (1)

Wherein C_t_ = net Zn trafficked and accumulated in the *C. elegans* after 24 h, and C_e_ = amount of RBITC@ZIF-8 MOF nanoparticles exposed to *C. elegans*.

## 3 Results and Discussion

### 3.1 Characterisation of ZIF-8 and RBITC@ZIF-8 MOF Nanoparticles

**FIGURE 1:**
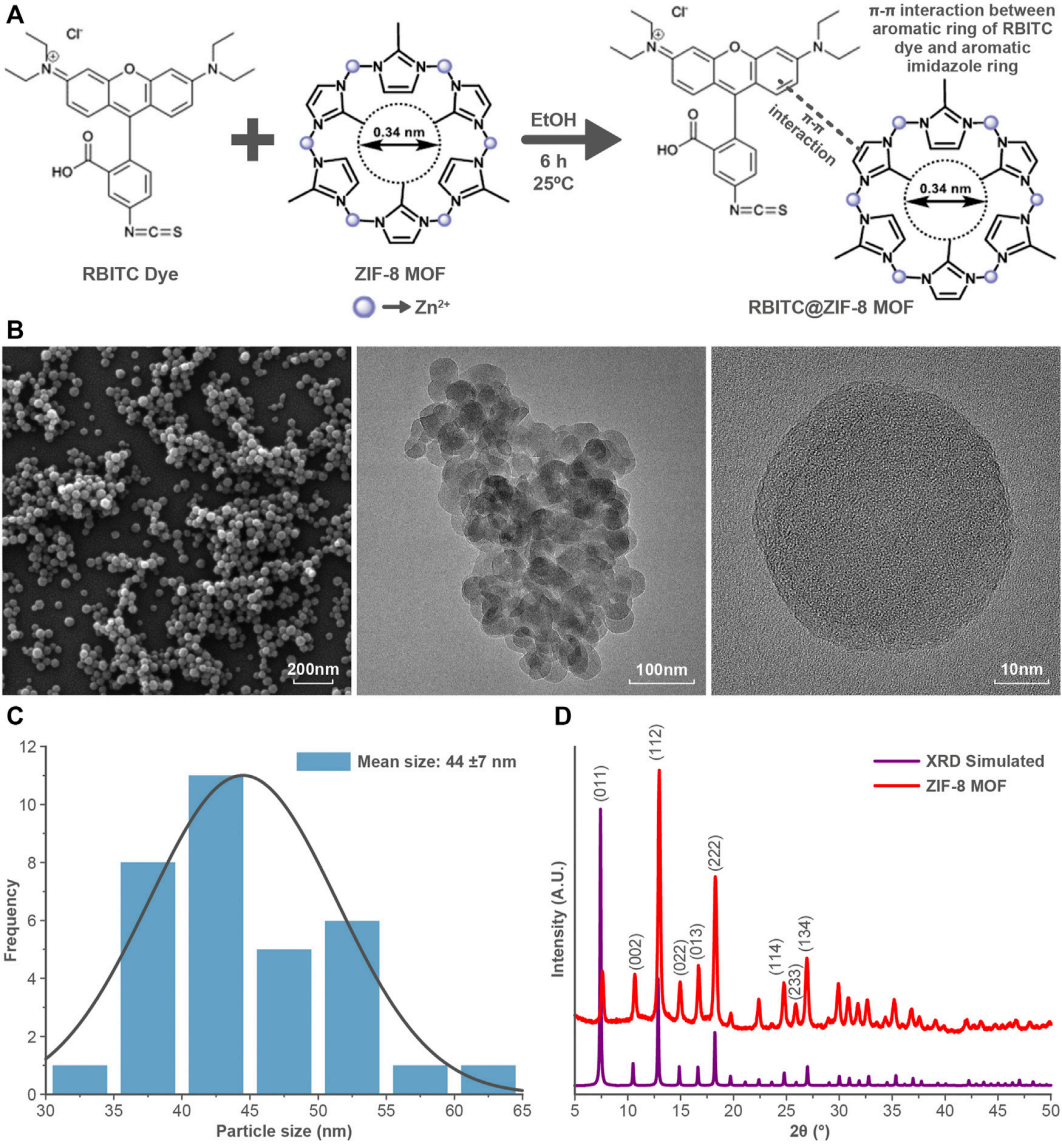
**(A)** Schematic showing the structure of ZIF-8 MOF nanoparticles and interaction of RBITC with ZIF-8 MOF nanoparticles. **(B)** SEM and TEM images of ZIF-8 MOF nanoparticles show the particle size and morphology. **(C)** Size distribution of ZIF-8 MOF nanoparticles. **(D)** X-ray diffraction pattern of ZIF-8 MOF nanoparticles indicates the phase purity of the nanoparticles.

ZIFs are mostly composed of a divalent metal ion such as Zn^2+^ or Co^2+^ linked to the nitrogen atom of the imidazole group and, hence forming a tetrahedral framework. Zinc imidazolate metal organic frameworks (ZIF-8 MOF) are composed of Zn^2+^ as a metal cation linked with the organic ligand 2-methylimidazole that forms interconnected large cavities of size 1.16 nm through the window of 0.34 nm (Figure 1A). ZIF-8 MOF nanoparticles were of rhombic dodecahedron shape and with a mean size of 44 ± 7 nm (Figures 1B,C) (Liu et al., 2017). The diffractogram pattern of ZIF-8 MOF nanoparticles confirms the presence of cubic centred lattice (CIF File No. 4118891), which agrees with published reports (Cravillon et al., 2009). The mechanism of attachment of RBITC on ZIF-8 MOF nanoparticles is shown in Figure 1A. In the presence of ethanol and heating, there is a π-π interaction between the aromatic ring of RBITC dye and the aromatic imidazole ring of ZIF-8. There was no difference in the XRD pattern between unlabelled ZIF-8 MOF nanoparticles and RBITC loaded ZIF-8 MOFs (Khan et al., 2020). The hydrodynamic diameters (d_nm_) of ZIF-8 MOF and RBITC@ZIF-8 MOF, measured using DLS, were found to be 717 and 1051 nm, respectively. Zeta potential measurements indicated both the variants of ZIF-8 MOF and RBITC@ZIF-8 MOF (ZIF-8 MOF: +13.13 mV; RBITC@ZIF-8 MOF: +4.08 mV) to be positively charged.

**FIGURE 2:**
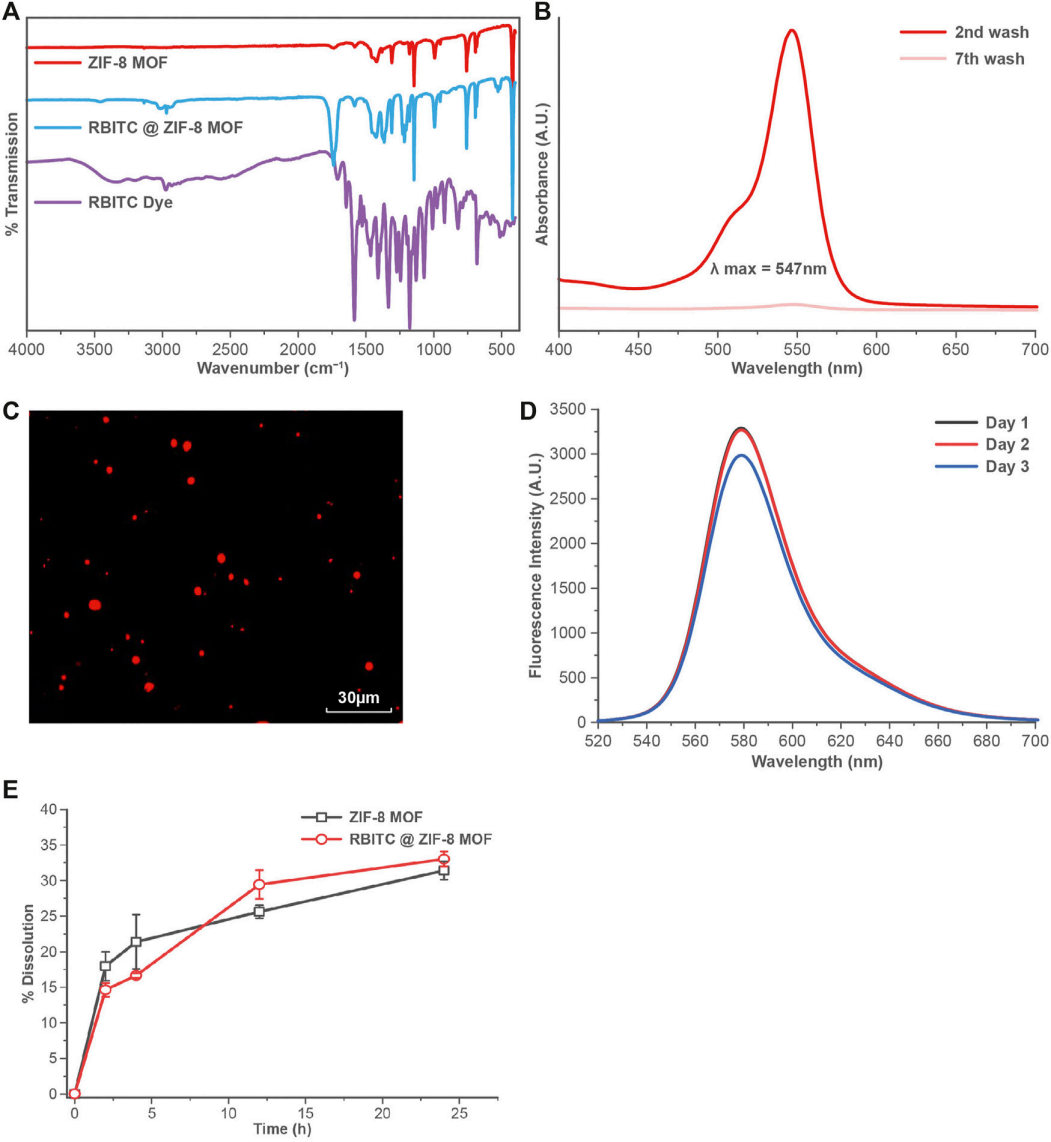
**(A)** FTIR analysis showing the presence of both RBITC and ZIF-8 MOFs characteristic peaks in the spectrum. **(B)** UV-Vis spectral scan showing the absence of RBITC spectra from the supernatant during the RBITC@ZIF-8 MOF nanoparticles washing process. **(C)** Fluorescent imaging of RBITC@ZIF-8 MOF nanoparticles upon excitation at 507 nm. **(D)** Fluorescent emission stability of RBITC@ZIF-8 MOF nanoparticles for up to 72 h. **(E)** Dissolution of RBITC@ZIF-8 MOF nanoparticles and ZIF-8 MOF nanoparticles in S-buffer (n = 3, data presented as mean ± standard deviation).

FTIR analysis of ZIF-8 MOF nanoparticles showed the characteristic peaks at 1145 cm^−1^, and 995 cm^−1^ corresponding to the C-H and C-C bond stretching of the aromatic ring (Figure 2A). The RBITC labelling on ZIF-8 MOF nanoparticles was confirmed by FTIR analysis. There are three major peaks of the RBITC dye present at 1180, 1337, and 1593 cm^−1^, which represent the C-O bond stretching from the carboxylic group and the C-N and N-H bending from the aromatic amine group, respectively. RBITC@ZIF-8 MOF nanoparticles were washed in deionised water several times and the UV-Vis spectrum of the supernatant was measured at several time points. Washing for up to 7 cycles ensured that the untagged dye is completely removed after the dye functionalization process (Figure 2B). The fluorescent RBITC@ZIF-8 MOF nanoparticles showed a uniform distribution (Figure 2C) when visualized using fluorescent microscopy. The fluorescence stability of RBITC@ZIF-8 MOF nanoparticles for 72 h in S-buffer was performed using Fluorescence spectrometry (*λ*_excitation_ = 507 nm; *λ*_emission_ = 568 nm). Figure 2D shows good stability of the fluorescence intensity from the MOF nanoparticles.

A time-dependent dissolution experiment was performed on the ZIF-8 MOF nanoparticles to measures the release of Zn^2+^. The dissolution was calculated in terms of the amount of Zn ions released in the S-buffer at various time intervals. It was observed that both pristine and RBITC@ZIF-8 MOF nanoparticles showed a similar dissolution profile, with up to 30% dissolution at the end of 24 h (Figure 2E). In contrast, we tested the dissolution of RBITC@ZIF-8 MOF nanoparticles in deionised water and the measured Zn release was ∼8.07 and 9.31% dissolution after the end of 4 and 12 h, respectively. The possible reason for an increase in the dissolution rate of RBITC@ZIF-8 MOF in S-buffer (high ionic strength (IS)) could be the instability of the organic framework of the ZIF-8 MOF. The interaction of organic ligands of ZIF-8 MOF with the salts in the S-buffer may have led to the detachment of surface organic species, followed by the release of Zn ions from the ZIF-8 MOF through an ion exchange mechanism (Yang et al., 2022). The dissolution data of ZIF-8 MOF nanoparticles indicate that a longer exposure time would lead to a significant release of Zn^2+^ in S-buffer. In our study, the exposure time of MOF nanoparticles while preparing the feeding mixture with S-buffer was about 30 s, which would have a minimum impact on the agglomeration/dissolution of the MOF nanoparticles. The dissolution is expected to create a minimal impact on the solubility and stability of RBITC@ZIF-8 MOF nanoparticles when exposed to S-buffer due to a short dispersion time (about 30 s to 1 min) before being exposed to worms. Therefore, it is expected that not all but a majority of RBITC@ZIF-8 MOF nanoparticles ingested by *C. elegans* were intact particles and not in their dissolved form.

### 3.2 *In Vivo* Studies

The nanoforms of Zn based materials (nanoparticles, MOFs) are used for environmental remediation applications and their excessive use could increase the environmental burden and may have an impact on the soil and sediment biota (Rajput et al., 2018). Such instances may be possible for ZIF-8 MOF nanoparticles as well, because of the nanoscale size effect. The MOFs can dissolves in biological media and interacts with the soil and sediments due to their complex chemical nature leading to its chemical and biological transformation. The value of predicted environmental concentration (PEC) of ZIF-8 MOFs nanoparticles is still a matter of investigation. However, there are existing reports on the PEC of ZnO nanomaterials in several environments such as wastewater, soil, and sediments, which could act as reference values. An estimation through modelling shows that the environmental concentration of ZnO nanoparticles ranges from 0.24 to 0.661 μg kg^−1^ in soil and sediments, which is currently showing an increasing trend going up to 1.82 μg kg^−1^ (Wu et al., 2019). It is not ideal to take the PEC values of ZnO nanoparticles as the basis for conducting a realistic uptake study for Zn MOF nanoparticles in worms. However, it could be a good starting point for conducting toxicity experiments.

**FIGURE 3:**
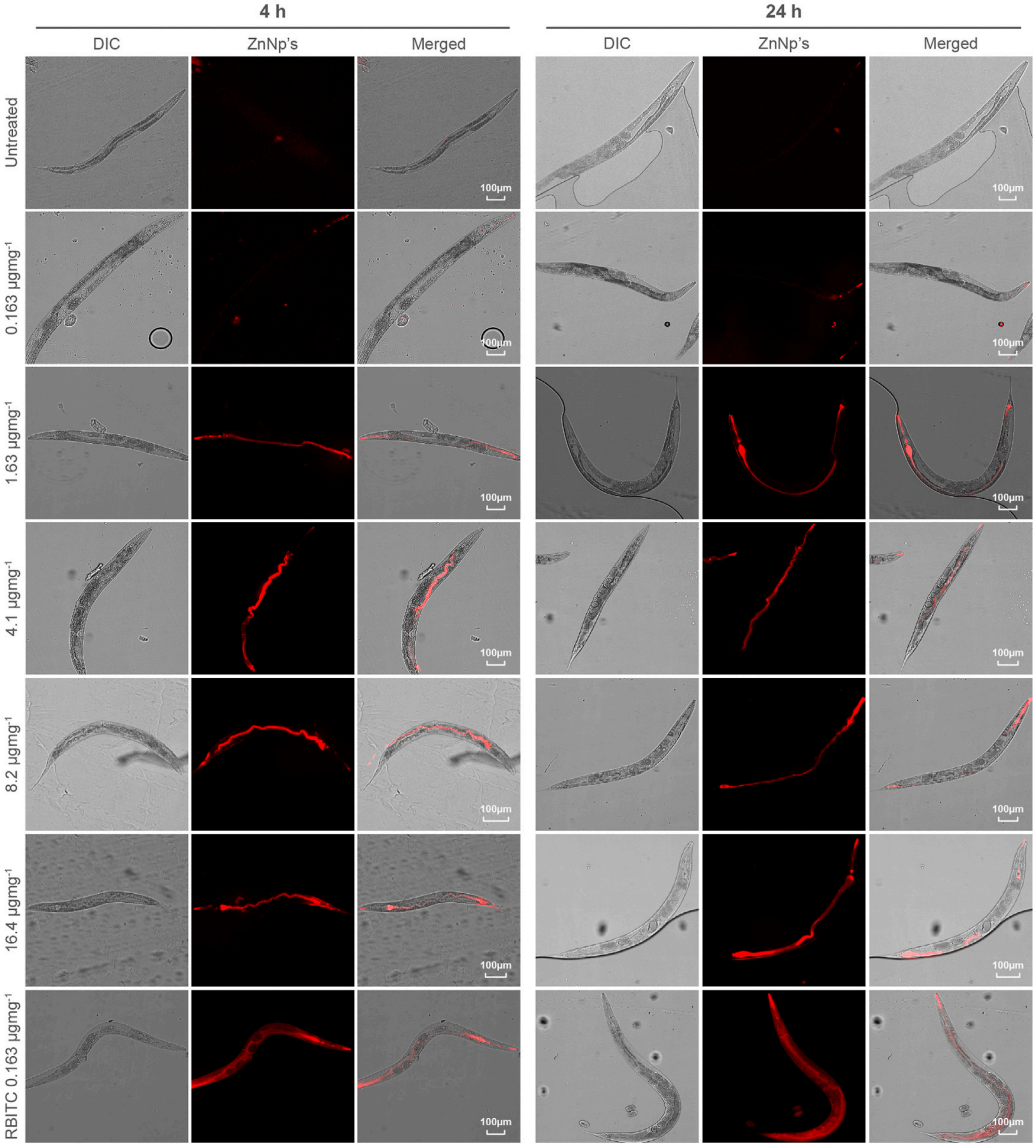
Tracing of accumulated RBITC@ZIF-8 MOF nanoparticles in *C. elegans*: DIC and epifluorescence images of untreated, RBITC@ZIF-8 MOF nanoparticles treated and only RBITC treated worms for 4 and 24 h. Ingested RBITC@ZIF-8 MOF nanoparticles were observed in the pharyngeal region and gut lumen. RBITC dye was absorbed by the worm but did not enter any organ region (Values are from three independent experiments and n = 10 for each experiment) (Scale bar = 100 μm).

*C. elegans* were exposed to RBITC@ZIF-8 MOF nanoparticles through a naturally feeding mechanism and were not force-fed. To trace the ingestion and distribution of MOF nanoparticles *in-vivo* (Figure 3), synchronized L4 stage worms were starved for 1 h and then treated with RBITC@ZIF-8 MOF nanoparticles at different concentrations (0.163 μg mg^−1^ −16.4 μg mg^−1^) for 4 and 24 h at 20°C. The exposure concentration of RBITC@ZIF-8 MOF corresponds to the mass of RBITC@ZIF-8 MOF per dry weight of *C. elegans* used in the study. Table 1 (Supplementary Material S1) shows the MOF exposure concentrations, and the equivalent Zn^2+^ exposure concentrations used in this study. Treated worms were transferred on agar plates and paralyzed with 1 mM Levamisole to ease the imaging process. Fluorescence was detected in pharyngeal and gut lumen regions of the worms after 4 h of treatment, for exposure concentrations above 0.163 μg mg^−1^. After 24 h of treatment, fluorescence was detected in all the exposure concentrations with enhanced fluorescence intensity. Interestingly, worms treated with higher concentrations of RBITC@ZIF-8 MOFs (≥1.63 μg mg^−1^) for 24 h showed a bright fluorescence signal in the tail regions, which shows the inability of the worm to excrete the RBITC@ZIF-8 MOF. It has been reported that when the worms are not provided with food, the process of excretion and defecation ceases. Also, studies demonstrated that in absence of food, the accumulation of nanoparticles increases in the worms, and they are retained for up to 24 h (Mohan et al., 2010; Le Trequesser et al., 2014). Therefore, there is a possibility that the RBITC@ZIF-8 MOFs nanoparticles were retained in the tail region for a longer duration and were not excreted up to 24 h. (Figure 3). To assess the stability of RBITC coupling to ZIF-8 MOFs after ingestion by the worm and to confirm the observed fluorescence signal in the intestinal region is of RBITC@ZIF-8 MOFs and not the dye alone, a control experiment was performed where worms treated with dye alone at a maximum concentration of 0.163 μg mg^−1^ for 4 h and 24 h. For both the timepoints, fluorescence was observed all over the body but failed to enter any organ regions. Together, these results suggest that the worms can ingest the labelled nanoparticles but face difficulty in excreting them from the digestive system.

**TABLE 1:** Summary of selected toxicological studies performed on ZIF-8 nanoparticles.

Type of MOF	Size	Organism model used	Major findings	Ref
ZIF-8 MOF	80±15 nm	Zebrafish embryos	ZIF-8 MOF provoked a significant decrease in embryo survival. ZIF-8 was toxic at 200 µM concentration (embryo viability at 120 hpf: 33.3%)	Ruyra et al., 2015
ZIF-8 MOF	60 nm	HeLa and J774 cell line	ZIF-8 MOF showed some cytotoxicity to HeLa and J774 cell lines, IC50 (HeLa) = 0.100 mg mL^−1^; (J774) = 0.025 mg mL^−1^	Tamames-Tabar et al. (2014)
ZIF-8 MOF	200 nm	Algae	ZIF-8 at a concentration of 0.01–1 mg L^−1^ induce significant algal growth inhibition, plasmolysis, membrane permeability, chloroplast damage, and chlorophyll biosynthesis, and the above alterations are recoverable	Zhang et al. (2021)
ZIF-8 MOF	80–200 nm	Corbicula fluminea	With exposure doses ranging from 0 to 50 mg L^−1^, ZIF-8 MOF induced oxidative stress behaviours similar to the hormesis effect in the tissues of C. fluminea. The oxidative stress induced by ZIF-8 MOF and the released Zn^2+^ was the crucial cause of the toxic effects	Yang et al. (2022)

**FIGURE 4:**
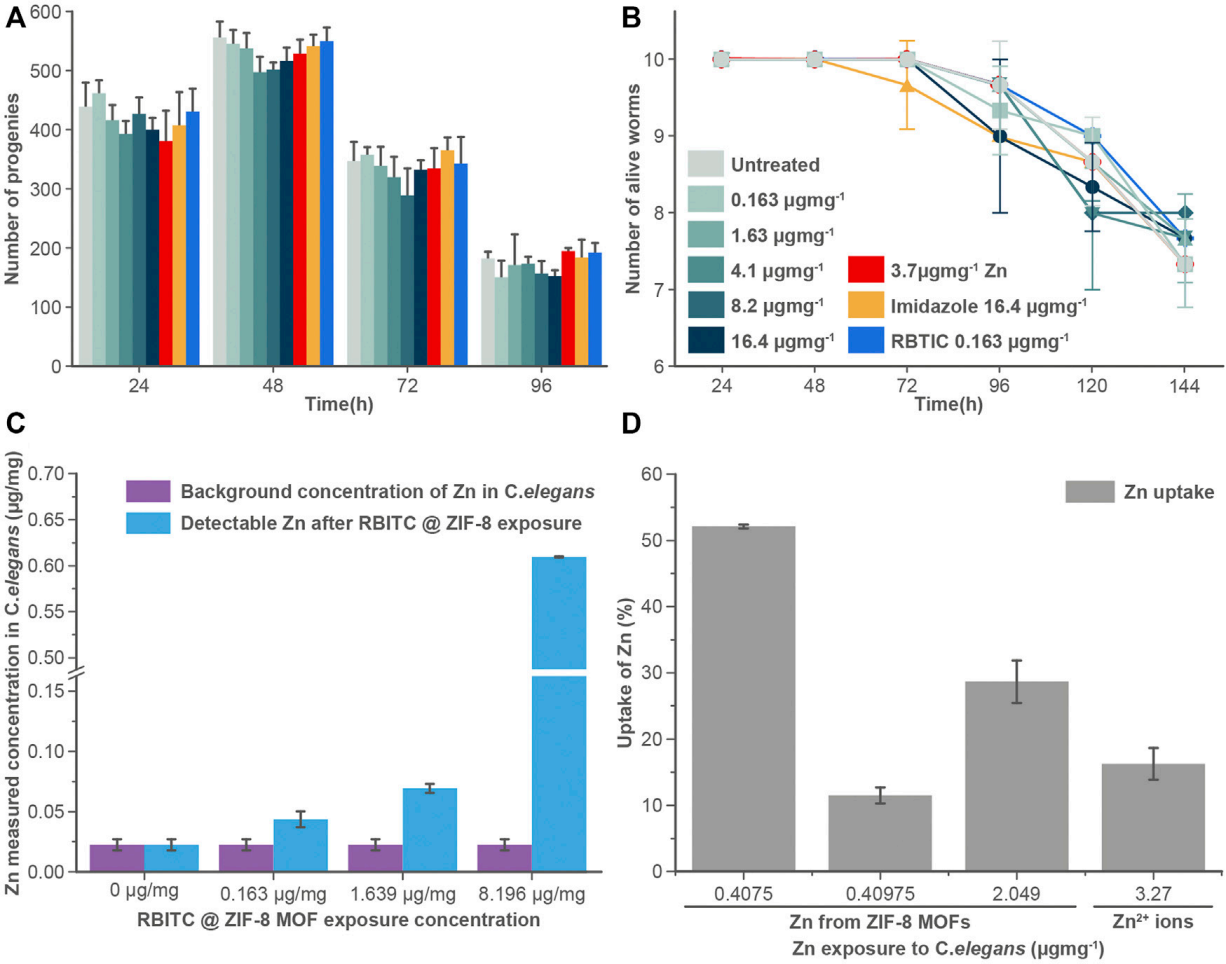
**(A)** Brood size and **(B)** longevity assay: RBITC@ZIF-8 MOF nanoparticles treated (0.16–16.4 μg mg^−1^), Zinc alone (3.27 μg mg^−1^) treated, imidazole treated (16.4 μg mg^−1^), and RBITC (1.64 μg mg^−1^) alone treated worms did not show a statistically significant difference in their egg-laying capacity or survival rate compared to untreated worms. Worms were treated for 4 h and transferred to NGM plate seeded with OP50. Values are from three independent experiments (N = 10 for each experiment). **(C)** Measured Zn concentration in *C. elegans* after exposure to RBITC@ZIF-8 MOF nanoparticles at exposure concentration (0.163–8.2 μg mg^−1^) **(D)** Percentage uptake of RBITC@ZIF-8 MOF nanoparticles after 24 h. The calculation were performed by substracting the background Zn in untreated control. (Data presented as mean ± standard deviation).

The uptake and accumulation of RBITC@ZIF-8 MOF in *C. elegans post-exposure* to a range of concentrations (0.163–8.2 μg mg^−1^) for 24 h were assessed by using ICP-MS. 24 h post-exposure to RBITC@ZIF-8 MOF, the *C. elegans* were digested in an ashing mixture (3:1 ratio of HNO_3_ and H_2_O_2_) to measure the concentration of Zn inside the worms. The background Zn present in *C. elegans* has been reported to be 0.09 ± 0.02, 0.10 ± 0.02, and 0.14 ± 0.03 μg mg^−1^ in L1, L3 and L5 stage of the worm life cycle respectively (Kumar et al., 2016). Based on our ICP-MS measurements, the background Zn content was found to be 0.02 μg mg^−1^. The low background concentration made it easy to measure the administered concentration of Zn upon exposure to RBITC@ZIF-8 MOF nanoparticles. On exposure to 0.163, 1.63, and 8.2 μg mg^−1^ of RBITC@ZIF-8 MOF nanoparticles, the net uptake of Zn was found to be 0.04 ± 0.006, 0.06 ± 0.003 and 0.6 ± 0.003 μg mg^−1^, respectively (Figure 4C). Since, Zn corresponds to 25% of the net weight of ZIF-8 MOF nanoparticles, the percentage uptake of RBITC@ZIF-8 MOF nanoparticles on exposure concentrations of 0.163, 1.63, 8.2 μg mg^−1^ of RBITC@ZIF-8 MOF nanoparticles was found to be 52.1, 11.4 and 28.6%, respectively (Figure 4D). We did not find any trend in the increase in uptake and accumulation of RBITC@ZIF-8 MOF nanoparticles with an increase in exposure concentration, which could be due to the natural feeding mechanism and the effect of the starvation period of each worm. We also treated the worms with Zn^2+^ (3.27 μg mg^−1^) and in that case, it was observed that 0.5 ± 0.002 μg mg^−1^ (16%) of Zn was taken up by the worms. Uptake studies indicate that owing to the relatively lower background concentration of Zn, it is easier to track RBITC@ZIF-8 MOF uptake even at an exposure concentration as low as 0.163 μg mg.^−1^

To understand the effect of ingested MOF nanoparticles on the reproductive capacity and longevity of worms (Figures 4A,B), *C. elegans* were treated with RBITC@ZIF-8 MOF nanoparticles (0.163–16.4 μg mg^−1^), Zn^2+^ (3.27 μg mg^−1^), imidazole (16.4 μg mg^−1^), and free dye RBITC (0.163 μg mg^−1^). The control set of experiments was performed on S-buffer treated worms. Time points analysis results from treated and untreated worms show that there is no significant difference in any of their behaviour. Our results show that RBITC@ZIF-8 MOF nanoparticles showed no significant toxicity under *in vivo* conditions in *C. elegans*. The existing results were comparable with the work reported by Zhang et al. (2021) on algae. In this work, the authors reported that the exposure of ZIF-8 MOFs at concentrations as low as 0.01–1 μg ml^−1^ can induce a significant level of inhibition of algal growth, chloroplast damage, membrane permeability, alteration in the biosynthesis of chlorophyll etc. However, the authors reported that these effects could be reversible. The adversity caused by ZIF-8 was reportedly weak as a majority of the effects were restored by the worms after some time (Zhang et al., 2021). In our case, the brood size and the life span of the worms didn’t show any change (post-exposure to RBITC@ZIF-8 MOF nanoparticles) as compared to the control. There is a possibility that the worms started excreting out the RBITC@ZIF-8 MOF nanoparticles after a certain time period once they were fed again. However, no imaging experiments were performed to show the excretion of the fluorescent labelled nanoparticles from the system.

With minimal toxicity of RBITC@ZIF-8 MOF nanoparticles under *in vivo* conditions, these fluorescent labelled MOF nanoparticles could be used to assess the biological translocation of MOFs, following exposure, within organisms. Even after 24 h of exposure, the particles were observed in the worm, which signifies the chemical stability of the RBITC@ZIF-8 MOF nanoparticles in the *in vivo* system. The uptake, distribution, excretion of the nanoparticles in *C. elegans* are reported in numerous reviews (Gonzalez-Moragas et al., 2015); however, MOF nanoparticles have a significantly different chemistry from inorganic nanoparticles, and therefore, their biological behavior requires an investigation. It was also observed that there was a decrease in hydrodynamic diameter (Supplementary Figure S1) of ZIF-8 MOF, this was because of sedimentation of the particles. As this work focuses on true toxicity of as synthesized ZIF-8 MOF nanoparticles, that’s why we did not used any capping agent to improve the colloidal stability of MOF. Though colloidal stability of particles is very much of interest in correlating the toxicity of particles, here in this study exposure and uptake of MOF by C.*elegans* would not get much affected by MOF stability. Further, the lack of colloidal stability of ZIF-8 MOF nanoparticles could significantly corresponds to their agglomeration and prolong accumulation within the tail region of the worms, which in return slowing down the process of excretion. Rojas et al. (2022) showed that about 0.4 and 0.9% of the total exposure concentration of MIL-127 and CIS@MIL-127 MOF nanoparticles respectively were excreted from the worms. It shows that the tendency of worms excreting MOF from their body could be possibly due to higher rate of agglomeration with in the body of worms. The summary of previously studied toxicological impact of ZIF-8 MOFs are summarized in Table 1.

## 4 Conclusion

MOFs have seen tremendous application in the area of environmental remediation which merits a need to study its toxicological impact within various environment compartments. In this study, ZIF-8 MOF nanoparticles were synthesised and its biological impact on *C. elegans* was performed. The ZIF-8 MOF nanoparticles were ingested by *C. elegans* and showed accumulation through spectroscopic measurement and through microscopic analysis within the worm. In order to perform *in vivo* imaging studies RBITC labelled ZIF-8 MOF nanoparticles were synthesised. The fluorescent label was observed to be stable for up to 72 h. The stability of RBITC@ZIF-8 MOF nanoparticles along with a low observed toxicity effect makes it an ideal candidate to be used to detect the biological translocation of MOFs through fluorescent imaging and quantification for their toxicological profiling even at a very low concentration.

## Data Availability

The original contributions presented in the study are included in the article/Supplementary Material, further inquiries can be directed to the corresponding authors.
