# Supplementary material for: Toxicological Impact and in Vivo Tracing of Rhodamine Functionalised ZIF-8 Nanoparticles
Source: Front Toxicol. 2022 Jul 1;4:917749. doi: 10.3389/ftox.2022.917749 (PMC9283923; doi:10.3389/ftox.2022.917749)
Supplement: Supplementary file 1 [file DataSheet1.docx]

**Supplementary information file**

**Table S1: Uptake of** RBITC@ZIF-8 MOF nanoparticles by *C. elegans.* The data is reported in terms of equivalent uptake of Zn^2+^ by the worms and were estimated by using ICP-MS.

| **MOF Exposure**  **(µg/mg)** | **Equivalent Zn^2+^ in MOF (µg/mg)** | **Zn^2+^ uptake in C.*elegans* (µg/mg)** |
| --- | --- | --- |
| 0.163 | 0.040 | 0.021 |
| 1.639 | 0.406 | 0.047 |
| 4.1 | 1.016 | Not tested |
| 8.196 | 2.032 | 0.587 |
| 16.4 | 4.067 | Not tested |

**Table S2** Summary of toxicological studies performed on ZIF-8 nanoparticles.

| **Type of MOF** | **Size** | **Organism model used** | **Major findings** | **Ref** |
| --- | --- | --- | --- | --- |
| ZIF-8 MOF | 80$\pm15$nm | Zebrafish embryos | ZIF-8 MOF provoked a significant decrease in embryo survival. ZIF-8 was toxic at 200µM concentration (embryo viability at 120 hpf: 33.3%) | Ruyra et al 2014 |
| ZIF-8 MOF | 60 nm | HeLa and J774 cell line | ZIF-8 MOF showed some cytotoxicity to HeLa and J774 cell lines, IC50 (HeLa) = 0.100 mg mL^−1^; (J774) = 0.025 mg mL^−1^ | Tamames-Tabar et al 2013 |
| ZIF-8 MOF | 200 nm | Algae | ZIF-8 at a concentration of 0.01–1 mg L^-1^ induce significant algal growth inhibition, plasmolysis, membrane permeability, chloroplast damage, and chlorophyll biosynthesis, and the above alterations are recoverable | Zhang et al 2021 |
| ZIF-8 MOF | 80-200 nm | Corbicula fluminea | With exposure doses ranging from 0 to 50 mg L^-1^, ZIF-8 MOF induced oxidative stress behaviours similar to the hormesis effect in the tissues of C. fluminea. The oxidative stress induced by ZIF-8 MOF and the released Zn^2+^ was the crucial cause of the toxic effects. | Yang et al 2022 |







***Figure S1.*** *Colloidal stability representing hydrodynamic size evolution of A) ZIF-8 MOF and B) RBITC@ZIF-8 MOF in deionized water and S-buffer.*
